# Preparation of an advanced intermediate for the synthesis of leustroducsins and phoslactomycins by heterocycloaddition

**DOI:** 10.3762/bjoc.18.143

**Published:** 2022-10-04

**Authors:** Anaïs Rousseau, Guillaume Vincent, Cyrille Kouklovsky

**Affiliations:** 1 Institut de Chimie Moléculaire et des Matériaux d’Orsay, Université Paris-Saclay, CNRS, F-91405 Orsay, France

**Keywords:** cycloaddition, organolithium, stereoselective, total synthesis

## Abstract

A convergent strategy for the synthesis of leustroducsins and phoslactomycins has been designed, relying on the synthesis and the coupling of three main fragments. The central fragment was synthesized via a regio-and stereoselective nitroso Diels–Alder reaction with an enol phosphate, followed by reductive cleavage of the phosphate to the ketone **11b**. Coupling studies of this fragment with the lactone fragment was accomplished in a stereoselective fashion through a vinyllithium intermediate. An advanced synthetic intermediate was then obtained after functional group transformation.

## Introduction

**Figure 1:**
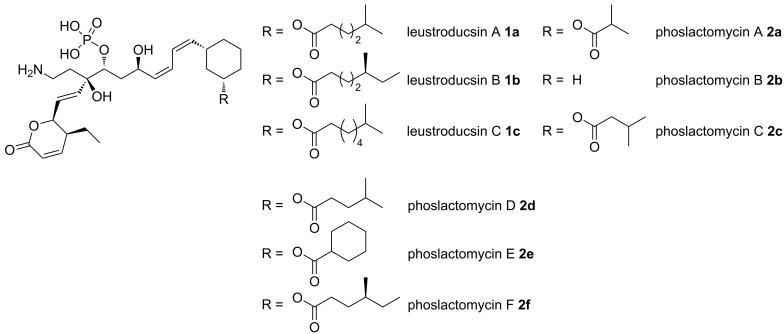
Structures of leustroducsins and phoslactomycins.

Leustroducsins **1a–c** and phoslactomycins **2a–f** are a family of closely related natural products extracted from *Streptomyces platensis* (leustroducsins) or *Streptomyces nigresens* (phoslactomycins) [1–4]. The main difference within this large family is the presence of an additional ester substituent on the terminal cyclohexane ring. Common structural motifs include a polyunsaturated acyclic chain with an unsaturated lactone ring and an amine-containing side chain (Figure 1).

These natural products have attracted much attention due to their original structure and to their activity as inhibitors of the serine/threonine phosphatase enzyme PP2A [5–6]. Therefore, phoslactomycins [7–12] and leustroducsins [13–17] have been subject of extensive synthetic studies.

**Figure 2:**
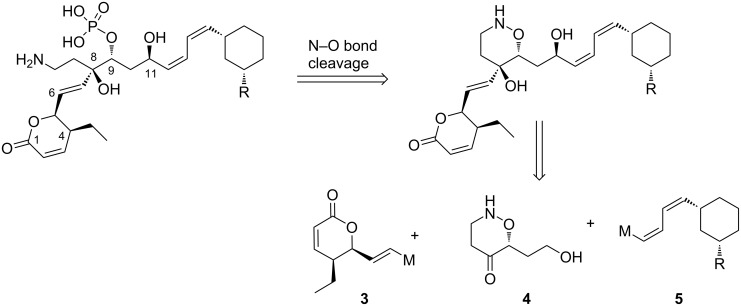
Synthetic strategy for the leustroducins and phoslactomycins.

In a project related to the synthesis of leustroducins and phoslactomycins, we have designed a convergent synthetic strategy involving the preparation and the coupling of three main fragments (Figure 2): the lactone fragment **3**, the central fragment **4** and the cyclohexane fragment **5**. We have previously described the enantioselective synthesis of the lactone fragment **3** [18]; we now disclose the synthesis of the oxazinone **4** and attempts for coupling both fragments for the synthesis of an advanced intermediate.

**Figure 3:**
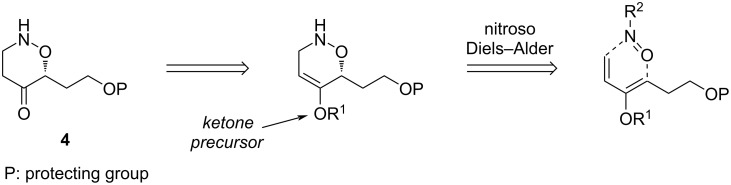
strategy for the synthesis of central fragment **4**: nitroso Diels–Alder reaction.

The synthetic strategy for the synthesis of the central fragment takes advantage of the proximity between the terminal amino function and the hydroxy function at C9. It was anticipated that both functions could arise from the cleavage of a N–O bond from an 1,2-oxazine, itself obtained by a nitroso Diels–Alder reaction from a chiral nitroso derivative and a functionalized diene (Figure 3). The nitroso Diels–Alder cycloaddition reaction has been well studied and has been used as a powerful tool for synthesis [19–22].

**Scheme 1:**
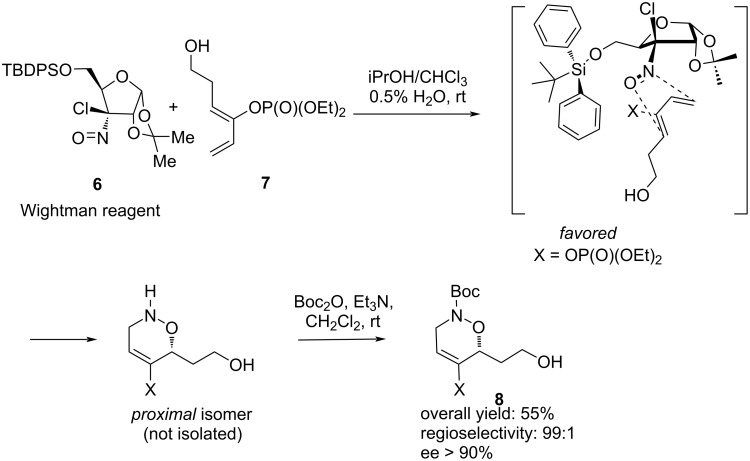
A highly regio-and stereoselective nitroso Diels–Alder cycloaddition between Wightman’s reagent **6** and a dienic enol phosphate.

We have reported extensive studies on the regio-and stereoselectivity of nitroso Diels–Alder reactions between various nitroso derivatives and functionalized dienes [23]. These studies led to the selection of enol phosphates as ketone precursors for the diene functionalization. Enol phosphates display several advantages over the related enol silyl ethers [24–25]: they are more stable towards acidic conditions, their electronic character contributes to high regioselectivity in cycloaddition reactions, and they can be converted to many other functions, including their hydrolysis to ketones [26]. In the other hand, we have shown that the Wightman reagent **6**, a chiral chloronitroso derivative [27], led to a complete regio- and stereoselective reaction with functionalized dienes (Scheme 1). The chiral auxiliary contributes to both regioselectivity and stereoselectivity. After hydrolysis of the chiral auxiliary and Boc-protection of the nitrogen atom, cycloadduct **8** was obtained in 55% yield and 90% ee. Therefore, the combination of both these reagents should provide a quick and selective access to the central fragment of leustroducsins/phoslactomycins.

## Results and Discussion

### Asymmetric cycloaddition

**Scheme 2:**
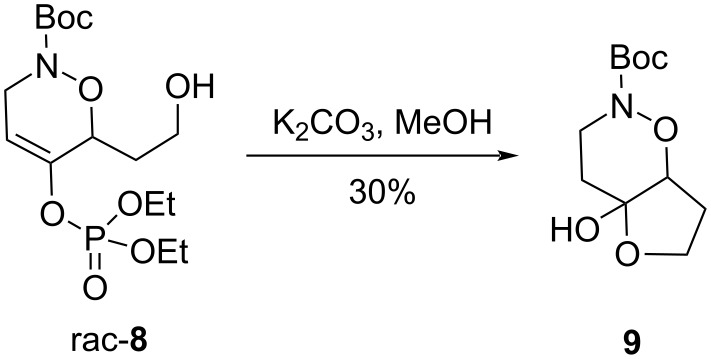
Hydrolysis of enol phosphate in the unprotected cycloadduct.

Preliminary studies for the conversion of enol phosphate to the corresponding ketone were accomplished using an unprotected primary alcohol. However, it appeared that hydroxy group protection was necessary: control experiments made on the racemic cycloadduct **8** showed that basic hydrolysis of the enol phosphate led to the cyclic hemiacetal **9** in modest yield (Scheme 2).

**Scheme 3:**
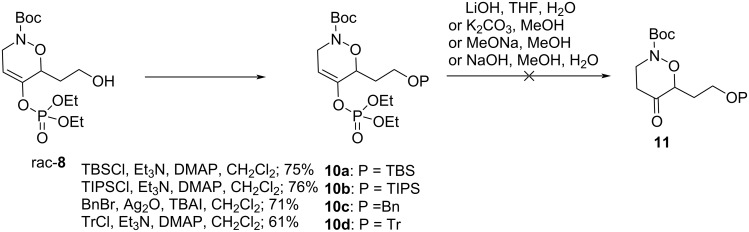
Attempts for hydrolysis of the enol phosphate under basic conditions.

Therefore, compound **8** was protected as silyl or benzyl ether using standard techniques. Unfortunately, no hydrolysis under several basic conditions provides the target ketone, no conversion and/or decomposition being observed (Scheme 3).

**Scheme 4:**
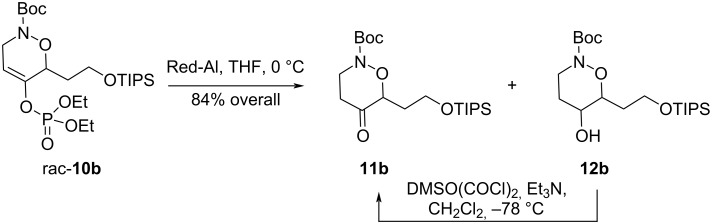
Cleavage of enol phosphate with Red-Al.

Enol phosphates can be hydrolysed under basic, acidic or reductive conditions [26]. Although acidic conditions could not be used due to the lability of the nitrogen Boc-protecting group, we found that the TIPS-protected cycloadduct **10b** could be cleanly transformed into the ketone **11b** with excess Red-Al [28], together with a small amount of the over reduced alcohol **12b**, which could be reoxidized to **11b** (Scheme 4). Other substrates failed to deliver appreciable yields of the ketone under the same conditions.

**Scheme 5:**
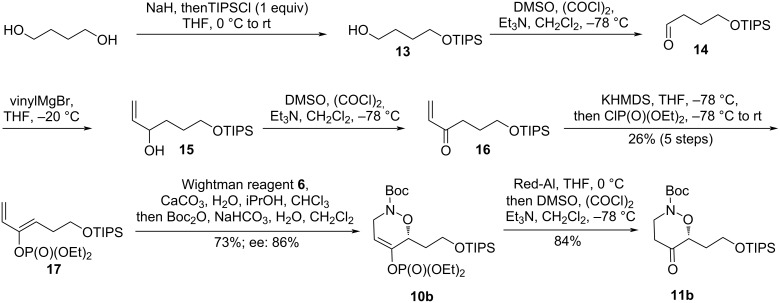
Synthesis of the protected central fragment **11b**.

These studies validate the role of TIPS ether as protecting group for the primary alcohol. At this stage we wondered whether it was possible to perform the whole synthetic sequence with this protecting group. Accordingly, the enol phosphate **13** was synthesized in five steps (26% overall yield) from 1,4-butanediol (Scheme 5). Since cycloaddition with the Wightman reagent **6** releases hydrogen chloride in the reaction medium, it was found necessary to add a small amount of calcium carbonate. Optically active cycloadduct **10b** was obtained in 73% yield and 86% ee after nitrogen protection as its Boc-carbamate. Ketone **11b** was obtained by Red-Al reduction in identical yield to the racemic equivalent.

We have therefore completed a quick, efficient and selective access to the central core of leustroducsins/phoslactomycins using an asymmetric nitroso Diels–Alder reaction. This fragment displays a ketone function that will be used for coupling with the lactone fragment **3** by generation of the tertiary alcohol.

### Studies in fragment coupling

**Scheme 6:**
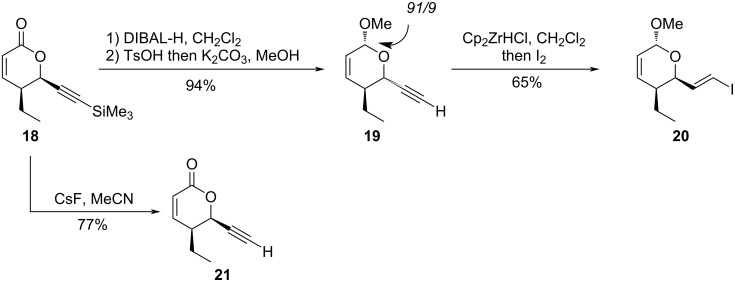
Synthesis and derivatization of the lactone fragment.

We have previously reported the synthesis of the lactone fragment by catalytic asymmetric [2 + 2] cycloaddition followed by ring extension [18]. The initial product was the TMS-acetylene **18** which could be easily desilylated to give **21**. However, model studies for coupling revealed the incompatibility of the lactone function; therefore, it was reduced with DIBAL-H then transformed into **19** by a one pot acetalization–desilylation procedure (91:9 mixture of diastereomers) [17]. Hydrozirconation followed by treatment with iodine furnished the target vinyl iodide **20** (Scheme 6); iodination with NIS, as previously described [29], gave lower yields.

**Scheme 7:**
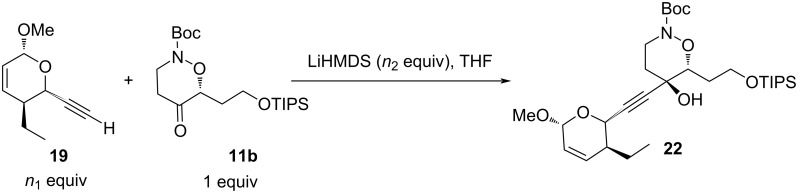
Coupling reaction between alkyne **19** and ketone **11b**.

We first attempted the coupling with the terminal alkyne **19**, anticipating the possibility of reducing the triple bond after coupling reaction. In agreement with literature precedents, we chose LiHMDS for deprotonation of **19** [30–31]. However, condensation of the corresponding lithium acetylide to the ketone **11b** gave modest and non-reproducible yields of the desired product **22** (Scheme 7, Table 1). The configuration of the newly created stereogenic center was undetermined.

**Table 1:** Coupling reaction between alkyne **19** and ketone **11b**.

entry	*n*_1_	*n*_2_	conditions	yield

1	1	1,2	−78 °C, 15 min, then rt, 8 h	21%
2	1	1,2	−78 °C, 2 h, then rt, 16 h	16%
3	1,5	1,8	−78 °C, 2 h, then rt, 3 h	24%
4	1,5	1,6	−78 °C, 2 h, then rt, 4 h	39%

These experiments showed the necessity to perform a fast reaction in order to avoid degradation. The optimal amount of base was found to be 1.6 equivalents (Table 1, entry 4). Higher amounts lowered the yields (Table 1, entry 3), probably due to competitive enolization of the cyclic ketone. Excess alkyne was also necessary, as low yields were obtained when using equimolar amounts of both **19** and **11b** (Table 1, entries 1 and 2).

**Scheme 8:**
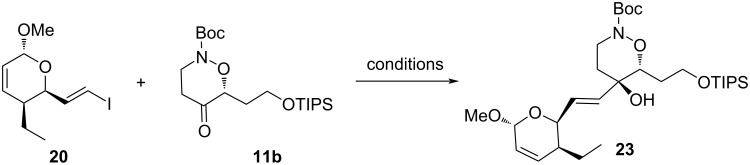
Coupling reaction between vinyl iodide **20** and ketone **11b**.

These disappointing results with alkyne **19** prompted us to investigate the coupling with an organometallic reagent derived from vinyl iodide **20**. This reagent was already synthesized and coupled with acyclic ketones in previous syntheses of leustroducsins or phoslactomycins [7–17]. Thus, treatment of **20** with *n*-butyllithium in THF gave the organometallic intermediate which was condensed onto ketone **11b** (Scheme 8, Table 2). Since no product was obtained under these standard conditions, we considered the use of additives in order to make the organolithium intermediate more nucleophilic. However, no reaction was observed when ZnMe_2_ (which was used in the synthesis of leustroducsin B by Trost and co-workers [17]) was added; trimethylaluminum and cerium chloride also failed to promote the reaction. However, switching the solvent from THF to toluene afforded 21% of product **23** with CeCl_3_ as additive. It appeared that the solvent had more influence on the course of the reaction than the metal. Indeed, reaction between vinyl iodide and ketone with *n*-BuLi in toluene [32] without any additive gave a reproducible 46% yield of **23**. Optimal conditions were obtained using 1.8 equivalents of vinyl iodide and 1.7 equivalents of BuLi (Table 2, entry 6).

**Table 2:** Coupling reaction between vinyl iodide **20** and ketone **11b**.

entry	conditions	yield

1	2 equiv **20**, 1.8 equiv *n*-BuLi, THF, −78 °C to rt	0%
2	2 equiv **20**, 1.8 equiv *n*-BuLi, 2 equiv ZnMe_2_, THF, −78 °C to rt	0%
3	2 equiv **20**, 1.8 equiv *n*-BuLi, 2 equiv AlMe_3_, THF, −78 °C to rt	0%
4	2 equiv **20**, 1.8 equiv *n*-BuLi, 2 equiv CeCl_3_, THF, −78 °C to rt	0%
5	2 equiv **20**, 1.8 equiv *n*-BuLi, 2 equiv CeCl_3_, **toluene**, −78 °C to rt	21%
6	1.8 equiv **20**, 1.7 equiv *n*-BuLi, **toluene**, −78 °C to rt	46%

**Scheme 9:**
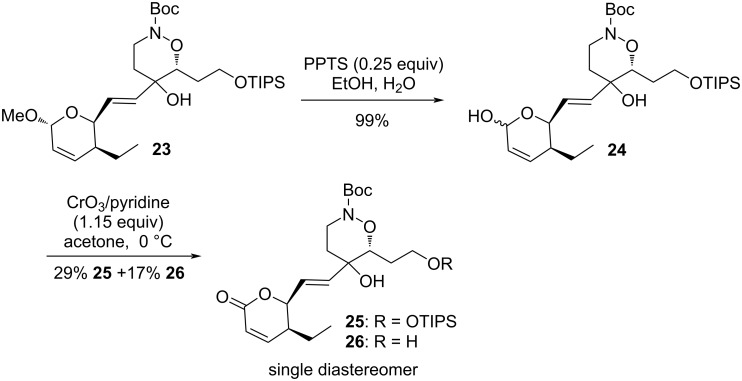
Oxidation of the acetal to the lactone.

It was difficult at this stage to determine the stereoselectivity of the coupling reaction since the starting acetal in **20** was a mixture of diastereomers. Therefore, we decided to oxidize the acetal in **23** to the corresponding lactone (Scheme 9). The acetal was first hydrolyzed to the hemiacetal **24** in quantitative yield. Oxidation of **24** proved delicate due to the lability of the tertiary allylic alcohol, and the presence of acid-sensitive protecting groups. Several conditions were tested: silver oxide on celite [33] failed to give any conversion. PCC with sodium acetate [34] gave only traces of the target lactone **25**. However, the use of the Jones’ reagent gave reproducible yields of **25**, together with the deprotected alcohol **26**. Under optimized conditions (1.15 equiv, 15 min) a combined 46% yield could be obtained. Higher equivalents of the oxidizing reagents or longer reaction time considerably lowered the yields.

NMR analysis of products **25** and **26** showed these compounds were obtained as single diastereomers, thus indicating the complete stereoselectivity of the coupling reaction. This validates the overall strategy for the synthesis of leustroducsins or phoslactomycins by the synthesis of a central cyclic core and its coupling with the other fragments.

## Conclusion

We have synthesized an advanced intermediate for the total synthesis of leustroducsins and phoslactomycins using a highly regio- and stereoselective nitroso Diels–Alder reaction, and a coupling reaction between a ketone and a vinyllithium reagent. This strategy offered quick and stereoselective access to an advanced precursor to these natural products. Further studies concerning the completion of the total synthesis via the preparation and coupling of the fragment **5** is under study in our laboratory.

## Experimental

Unless otherwise stated, all reactions were conducted in oven-dried glassware under an atmosphere of dry argon. Tetrahydrofuran was distilled over sodium/benzophenone ketyl under argon. Acetonitrile, dichloromethane, DMSO, DMF and toluene were distilled over calcium hydride under argon. All other reagents were used as received. Chromatographic purifications refer to flash chromatography on silica gel. ^1^H NMR spectra were measured at 250, 300, 360 or 400 MHz using CDCl_3_ as solvent using residual chloroform (7.26 ppm) as an internal reference. ^13^C NMR spectra were measured at 62.5, 75 or 90 MHz using residual chloroform (77.1 ppm) as an internal reference. High-resolution mass spectrometry (HRMS) analyses were conducted with electro spray ionization (ESI).

**6-Triisopropylsilyloxyhex-1-en-3-one (16):** A solution of oxalyl chloride (0.49 mL, 5.75 mmol, 1.5 equiv) in dichloromethane (12 mL) was cooled to −78 °C and DMSO (0.82 mL, 11.49 mmol, 3 equiv) was added over 5 min. After 15 min, a solution of the alcohol **15** [35] (1.044 g, 3.83 mmol) in dichloromethane (5 mL) was added over 5 min. The reaction mixture was stirred for 30 min at −78 °C before addition of triethylamine (2.7 mL, 19.15 mmol, 5 equiv). The cooling bath was removed and the solution was allowed to warm to rt in 30 min. It was then poured into diethyl ether (50 mL) and the solution was successively washed with saturated aqueous CuSO_4_ solution (4 × 12.5 mL), saturated aqueous NH_4_Cl solution (3 × 12.5 mL), dried (MgSO_4_), filtered and concentred under reduced pressure to give a brown oil (1.021 g, 99%). *R*_f_: 0,59 (10% AcOEt/cyclohexane); ^1^H NMR (300 MHz, CDCl_3_) δ 6.32 (dd*, J =* 17.7, 10.2 Hz, 1H), 6.19 (dd*, J =* 17.7, 1.5 Hz, 1H), 5.78 (dd*, J =* 10.2, 1.5 Hz, 1H), 3.68 (t*, J =* 6 Hz, 2H), 2.68 (t*, J =* 7.2 Hz, 2H), 1.86–1.77 (m*,* 2H), 1.00 (m*,* 21H) ppm; ^13^C NMR (75 MHz, CDCl_3_) δ 200.8, 136.7, 127.9, 62.4, 35.9, 27.2, 18.0, 12.0 ppm; HRMS (*m*/*z*): [M + Na]^+^ calcd 293.1907; found, 293.1898.

**(3*Z*)-3-Diethylphosphoryloxy-6-triisopropylsilyloxyhexa-1,3-dien (17):** A 0.5 M solution of potassium hexamethyldisilazide in toluene (4.4 mL, 2.22 mmol, 1.2 equiv) was added to a cooled (−78 °C) solution of diethyl chlorophosphate (0.27 mL, 1.85 mmol) in anhydrous THF (7 mL). A solution of the enone **16** (500 mg, 1.85 mmol) in THF (6 mL) was then slowly added. The solution was stirred 30 min at −78 °C, then 1 h at 0 °C and then 1 h at rt, before being poured in diethyl ether (35 mL). The solution was washed with 5% aqueous ammonia solution (18 mL). The aqueous layer was extracted with diethyl ether (3 × 35 mL) and the combined organic layers were dried (MgSO_4_), filtered and concentred under reduced pressure to give a brown oil. Purification by column chromatography (25% AcOEt/cyclohexane) gave the enol phosphate **17** as a yellow oil (200 mg, 26%). *R*_f_: 0.47 (30% AcOEt/cyclohexane); ^1^H NMR (360 MHz, CDCl_3_) δ 6.15 (dd*, J =* 17.3, 10.8 Hz, 1H), 5.47 (d*, J =* 17.3 Hz, 1H), 5.29 (dt*, J =* 7.2, 1.4 Hz, 1H), 5.08 (d*, J =* 10.8 Hz, 1H), 4.15–4.12 (m, 4H), 3.71 (t*, J =* 6.5 Hz, 2H), 2.48 (2dt*, J =* 7.2, 6.5 Hz, 2H), 1.31 (dt*, J =* 6.8, 1.1 Hz, 6H), 1.01 (m*,* 21H) ppm; ^13^C NMR (90 MHz, CDCl_3_) δ 146.2, 131.9, 118.0, 114.2, 64.4, 62.4, 30.0, 18.0, 16.2, 12.0 ppm; HRMS (*m*/*z*): [M + H]^+^ calcd 407.2377; found, 407.2359.

**(6*R*)-*tert*-Butyl 5-(diethoxyphosphoryloxy)-6-(2-((triisopropylsilyl)oxy)ethyl)-3,6-dihydro-2*H*-1,2-oxazine-2-carboxylate (10b):** A solution of the enol phosphate **17** (420 mg, 1.03 mmol) in chloroform (1.8 mL) was added to a solution of the Wightman reagent **6** (981 mg, 2.06 mmol, 2 equiv), calcium carbonate (206 mg, 2.06 mmol, 2 equiv) and water (40 µL, 2.06 mmol, 2 equiv) in isopropanol (1.8 mL). The mixture was stirred at rt for 30 h. Water (0.75 mL) was added and the solution stirred for additional 1 h. The pH was adjusted to 8 by addition of saturated aqueous NaHCO_3_ solution (1,6 mL), and a solution of Boc_2_O (899 mg, 4.12 mmol, 4 equiv) in chloroform (0.8 mL) was added. The solution was stirred at rt for 64 h and poured into a mixture of water (37 mL) and dichloromethane (74 mL); the layers were separated and the aqueous layer extracted with dichloromethane (3 × 74 mL). The combined organic layers were dried (MgSO_4_), filtered and concentred under reduced pressure. Purification of the crude product by column chromatography (30% AcOEt/cyclohexane) gave the cycloadduct **10b** as a yellow oil (404 mg, 73%). *R*_f_: 0.42 (30% AcOEt/cyclohexane); ^1^H NMR (360 MHz, CDCl_3_) δ 5.69 (m, 1H), 4.57 (broad d*, J =* 9.4 Hz, 1H), 4.16 (q*, J =* 7.2 Hz, 4H), 4.12–4.00 (m*,* 2H), 3.99–3.82 (m*,* 2H), 2.03–1.85 (m*,* 2H), 1.47 (s*,* 9H), 1.34 (t*, J =* 7.2 Hz, 6H), 1.05 (m*,* 21H) ppm; ^13^C NMR (90 MHz, CDCl_3_) δ 154.8, 146.8, 105.1, 81.7, 75.2, 64.8, 59.3, 43.7, 33.7, 28.4, 18.1, 16.2, 12.1 ppm; HRMS (*m*/*z*): [M + Na]^+^ calcd 560.2779; found, 560.2775; [α]^20^_D_ : +5.8 (*c* 1.0, CH_2_Cl_2_); ee: 8% (Whelk-O1, 1 mL/min, 95:5 hexane/EtOH, *t*_r_ (*R*) = 14.9 min, *t*_r_ (*S*) = 16.2 min).

**(6*R*)-*tert*-Butyl 5-oxo-6-(2-((triisopropylsilyl)oxy)ethyl)-1,2-oxazinane-2-carboxylate (11b):** A solution of the cycloadduct **10b** (404 mg, 0.751 mmol) in anhydrous THF (12 mL) was cooled to 0 °C and a 3 M solution of Red-Al**^®^** in toluene (1 mL, 3 mmol, 4 equiv) was rapidly added. After stirring 30 min at 0 °C, the reaction was hydrolyzed by addition of an saturated aqueous NH_4_Cl solution (4 mL). The solution was concentred under reduced pressurre, the residue taken up with dichloromethane (10 mL) and filtered, washing with dichloromethane (3 × 5 mL). The filtrate was concentred under reduced pressure to give a yellow oil (300 mg) consisting in a mixture of the ketone **11b** and the over-reduced alcohol. This mixture was carried into the next step without further purification.

DMSO (0.16 mL, 2.241 mmol, 3 equiv) was added dropwide to a cooled (−78 °C) solution of oxalyl chloride (0.1 mL, 1.121 mmol, 1.5 equiv) in dichloromethane (3.4 mL). After stirring 15 min at −78 °C, a solution of the crude product from reduction reaction (300 mg) in dichloromethane (2 mL) was added dropwise. After 30 min at −78 °C, triethylamine (0.52 mL, 3.735 mmol, 5 equiv) was added. The colling bath was removed and the solution stirred at rt for 40 min, before being poured into diethyl ether (45 mL). The solution was succesively washed with saturated aqueous CuSO_4_ solution (4 × 10 mL) and saturated aqueous NH_4_Cl solution (3 × 10 mL), then dried (MgSO_4_), filtered and concentred under reduced pressure. The residue was purified by filtration through a short plug of silica gel, eluting with ethyl acetate. Concentration under reduced pressure gave the pure ketone **11b** as an orange oil (255 mg, 84% over two steps). *R*_f_: 0.29 (10% AcOEt/cyclohexane); ^1^H NMR (250 MHz, CDCl_3_) δ 4.49 (dd*, J =* 8.3, 3.8 Hz, 1H), 4.18–4.08 (m*,* 1H), 3.94 (m*,* 4H), 2.67 (t*, J =* 7.0 Hz, 2H), 2.16–2.03 (m, 1H), 1.99–1.85 (m*,* 1H), 1.51 (s*,* 9H), 1.05 (m*,* 21H) ppm; ^13^C NMR (90 MHz, CDCl_3_) δ 206.6, 154.9, 85.1, 82.3, 59.0, 45.0, 36.5, 32.2, 28.4, 18.1, 12.1 ppm; HRMS (*m*/*z*): [M + Na]^+^ calcd 424.2490; found, 424.2480; [α]_D_^20^ +37.4 (*c* 0.5, CH_2_Cl_2_).

**(5*S*,6*R*)-5-Ethyl-6-ethynyl-5,6-dihydro-2*H*-pyran-2-one (21):** Caesium fluoride (290 mg, 1.91 mmol, 1.3 equiv) was added to a solution of the lactone **18** [6] (327 mg, 1.47 mmol) in anhydrous acetonitrile (15 mL). The solution was stirred at rt; after 2 h 20 min, additional CsF (112 mg, 0.74 mmol, 0.5 equiv) was added. After a total time of 3 h 30 min, the solution was partioned between diethyl ether (70 mL) and water (35 mL). The layers were separated, the organic layer was washed with saturated aqueous NaCl solution (35 mL). The combined aqueous layers were extracted with diethyl ether (2 × 70 mL). The organic layers were combined, dried (MgSO_4_), filtered and concentred under reduced pressure. Purification of the residue by column chromatography (25% Et_2_O/pentane) gave **21** as a yellow oil (171 mg, 77%). *R*_f_: 0.33 (30% Et_2_O/pentane); ^1^H NMR (250 MHz, CDCl_3_) δ 6.79 (dd*, J =* 9.8, 3.5 Hz, 1H), 6.05 (dd*, J =* 10.0, 2.0 Hz, 1H), 5.16 (dd*, J =* 4.8, 2.3 Hz, 1H), 2.68–2.59 (m*,* 1H), 2.56 (d*, J =* 2.0 Hz, 1H), 1.86–1.62 (m*,* 2H), 1.04 (t*, J =* 7.3 Hz, 3H) ppm; ^13^C NMR (90 MHz, CDCl_3_) δ 162.5, 148.8, 120.3, 77.4, 76.6, 70.7, 38.7, 22.6, 10.9 ppm; HRMS (*m*/*z*): [M + Na]^+^ calcd 173.0573; found, 173.0572; [α]_D_^20^ +132.0 (*c* 1.0, CH_2_Cl_2_).

**(2*R*,3*S*,6*RS*)-3-Ethyl-2-ethynyl-6-methoxy-3,6-dihydro-2*H*-pyran (19):** This compound was prepared according to reference [18].

A solution of the lactone **18** (1.23 g, 5.53 mmol) in anhydrous dichloromethane (10 mL) was cooled to −78 °C and a solution of DIBAL-H in toluene (1.2 M, 6 mL, 7.19 mmol, 1.3 equiv) was added dropwise. The reaction mixture was stirred at −78 °C for 30 min then poured into a NaHCO_3_ solution (5 mL). The layers were separated and the aqueous layer extracted with ethyl acetate (3 × 10 mL). The combined organic layers were dried (MgSO_4_), filtered and concentred under reduced pressure. The residue (1.3 g) was redissolved in anhydrous methanol (25 mL) and paratoluenesulfonic acid hydrate (53 mg, 0.277 mmol, 0.05 equiv) was added. After stirring 1 h at rt, solid K_2_CO_3_ (1.53 g, 11.06 mmol, 2 equiv) was added and the mixture stirred overnight at rt. Diethyl ether (50 mL) was added and the solution washed with water (2 × 50 mL). The organic layer was dried (MgSO_4_), filtered and carefully concentred under reduced pressure. Purification by column chromatography (5% Et_2_O/pentane), gave **19** as a colourless oil (866 mg, 94%, 91/9 mixture of stereoisomers). Analytical data were in agreement with literature data [18].

**(2*R*,3*S*,6*RS*)-3-Ethyl-2-((*E*)-2-iodovinyl)-6-methoxy-3,6-dihydro-2*H*-pyran (20):** This compound was prepared according to reference [18].

A solution of the alkyne **19** (300 mg, 1.80 mmol) dans in anhydrous dichloromethane (4.2 mL) was added dropwise to a suspension of Cp_2_ZrHCl (696 mg, 2.70 mmol, 1.5 equiv) in anhydrous dichloromethane (9 mL). After stirring at rt for 15 min, a solution of iodine (777 mg, 3.06 mmol, 1.7 equiv) in anhydrous dichloromethane (9 mL) was added dropwise until a light brown solution was obtained. The reaction mixture was hydrolyzed by successive addition of a saturated aqueous Na_2_S_2_O_3_ solution (25 mL) and water (9 mL). The layers were separated and the organic layer was washed with water (9 mL). The combined aqueous layers were back-extracted with diethyl ether (2 × 40 mL). The combined organic layers were dried (MgSO_4_), filtered and concentred under reduced pressure. Purification of the residue by column chromatography (2.5% Et_2_O/pentane) gave **20** as a yellowish oil (347 mg, 65%, 91:9 mixture of steroisomers). Analytical data were in agreement with literature data [18].

**Coupling reaction between vinyl iodide 20 and ketone 11b:** A solution of the vinyl iodide **20** (283 mg, 0.962 mmol, 1.8 equiv) in anhydrous toluene (2 mL) was cooled to −78 °C, and a *n*-butyllithium solution (2.3 M in hexanes, 0.39 mL, 0.909 mmol, 1.7 equiv) was added dropwise. The solution was stirred for 30 min at −78 °C then a solution of ketone **11b** (215 mg, 0.535 mmol, 1 equiv) in toluene (3.8 mL) was slowly added. The reaction was stirred at −78 °C for 45 h than slowly warmed to rt over 20 h. The reaction as quenched by addition of a saturated aqueous NH_4_Cl solution (3.8 mL). The layers were separated and the aqueous layer extracted with ethyl acetate (2 × 8 mL) and diethyl ether (2 × 8 mL). The combined organic layers were dried (MgSO_4_), filtered and concentred under reduced pressure. Purification of the residue by column chromatography (20 to 30% AcOEt/cyclohexane) gave the coupling product **23** as an orange oil, which was carried into the next step without further characterization (140 mg, 46%).

Product **23** was redissolved in 96% EtOH (3.9 mL) and pyridinium *para*-toluenesulfonate (17 mg, 0.066 mmol, 0.25 equiv) was added. The reaction mixture was stirred at rt for 24 h then neutralized by addition of a few drops of a saturated sodium hydrogen carbonate solution. The solvents were removed under reduced pressure and the residue partioned between ethyl acetate (5 mL) and water (2.5 mL). The layers were separated and the aqueous layer extracted with ethyl acetate (3 × 5 mL) and diethyl ether (5 mL). The combined organic layers were dried (MgSO_4_), filtered and concentred under reduced pressure to give the crude lactol **24** which was immediately engaged into the next reaction.

A solution of the above lactol (147 mg, 0.265 mmol) in acetone (6 mL) was cooled to 0 °C and a solution of the Jones reagent (2.2 M in water, 0.14 mL, 0.31 mmol, 1.15 equiv) was added. After stirring 15 min at 0 °C, the reaction was quenched by addition of a saturated aqueous sodium hydrogen carbonate solution (9 mL) and isopropanol (1.5 mL). The solvents were removed under reduced pressure and the residue partioned between ethyl acetate (11 mL) and water (5.5 mL). The layers were separated and the aqueous layer extracted with ethyl acetate (2 × 11 mL) and diethyl ether (2 × 11 mL). The combined organic layers were dried (MgSO_4_), filtered and concentred under reduced pressure. Purification of the residue by column chromatography (25 to 40% AcOEt/cyclohexane) gave first the protected lactone **25** as a sticky yellow oil (42 mg, 29% over two steps), further elution with 100% AcOEt gave the unprotected alcohol **26** (18 mg, 17%).

***tert*-Butyl (6*R*)-5-((*E*)-2-((2*S*,3*S*)-3-ethyl-6-oxo-3,6-dihydro-2*H*-pyran-2-yl)vinyl)-5-hydroxy-6-(2-((triisopropylsilyl)oxy)ethyl)-1,2-oxazinane-2-carboxylate (25):** Data for **25**: *R*_f_: 0.10 (30% AcOEt/cyclohexane); ^1^H NMR (360 MHz, CDCl_3_) δ 6.97 (dd*, J =* 9.7, 5.5 Hz, 1H), 6.05 (d*, J =* 9.7 Hz, 1H), 5.95 (dd*, J =* 15.5, 4.2 Hz, 1H), 5.82 (dd*, J =* 15.5, 1.4 Hz, 1H), 5.02 (ddd, app td*, J =* 4.2, 4.2, 1.4 Hz, 1H), 3.99–3.90 (m*,* 3H), 3.76–3.69 (m*,* 1H), 3.55 (td*, J =* 13.1, 2.7 Hz, 1H), 2.44–2.37 (m*,* 1H), 1.90–1.70 (m*,* 2H), 1.67–1.57 (m*,* 3H), 1.49 (s*,* 9H), 1.45–1.39 (m*,* 1H), 1.05 (m*,* 21H), 0.93 (t*, J =* 7.5 Hz, 3H) ppm; ^13^C NMR (62,5 MHz, CDCl_3_) δ 163.9, 155.1, 150.1, 135.5, 125.1, 121.0, 82.6, 81.8, 79.8, 70.8, 59.0, 42.3, 39.4, 35.9, 31.3, 28.4, 21.8, 18.1,12.0,11.1 ppm; HRMS (*m*/*z*): [M + Na]^+^ calcd 576.3327; found, 576.3330; [α]_D_^20^ +86.3 (*c* 1.1, CH_2_Cl_2_).

***tert*-Butyl (6*R*)-5-((*E*)-2-((2*S*,3*S*)-3-ethyl-6-oxo-3,6-dihydro-2*H*-pyran-2-yl)vinyl)-5-hydroxy-6-(2-hydroxyethyl)-1,2-oxazinane-2-carboxylate (26):** Data for **26**: *R*_f_: 0.38 (80% AcOEt/cyclohexane); ^1^H NMR (400 MHz, acetone-*d*_6_) δ 7.09 (dd*, J =* 10.0, 5.2 Hz, 1H), 6.02 (dd*, J =* 15.6, 5.5 Hz, 1H), 5.97 (dd*, J =* 10.0, 1.2 Hz, 1H), 5.85 (dd*, J =* 15.6, 1.2 Hz, 1H), 5.06 (ddd*, J =* 5.5, 4.0,1.2 Hz, 1H), 4.27 (s*,* exchangeable with D_2_O, 1H), 3.96–3.91 (m*,* 2H), 3.74–3.66 (m*,* 2H), 3.63–3.59 (m*,* 1H), 2.61–2.53 (m*,* 1H), 1.95–1.83 (m*,* 2H), 1.73–1.67 (m*,* 1H), 1.67–1.55 (m*,* 2H), 1.49 (s*,* 9H), 1.47–1.38 (m*,* 1H), 0.94 (t*, J =* 7.6 Hz, 3H) ppm; ^13^C NMR (100 MHz, acetone-*d*_6_) δ 163.83, 156.0, 151.0, 137.2, 126.1, 121.2, 84.0, 81.8, 80.7, 71.1, 59.5, 42.7, 40.0, 36.3, 31.3, 28.4, 22.2, 11.2 ppm; HRMS (*m*/*z*): [M + Na]^+^ calcd 420.1993; found, 420.1970.
